# Understanding LrgAB Regulation of *Streptococcus mutans* Metabolism

**DOI:** 10.3389/fmicb.2020.02119

**Published:** 2020-09-03

**Authors:** Sang-Joon Ahn, William Hull, Shailja Desai, Kelly C. Rice, David Culp

**Affiliations:** ^1^Department of Oral Biology, College of Dentistry, University of Florida, Gainesville, FL, United States; ^2^Department of Microbiology and Cell Science, Institute of Food and Agricultural Sciences, University of Florida, Gainesville, FL, United States

**Keywords:** *Streptococcus mutans*, pyruvate, LrgAB, dual-species, caries, mouse model

## Abstract

Lack of LrgAB renders cariogenic *Streptococcus mutans* more sensitive to oxidative stress, as well as limits the capacity of this organism to re-uptake pyruvate upon starvation. This study was aimed at investigating the ecological and metabolic contribution of LrgAB to competitive fitness, using *S. mutans* strains, that either lack or overexpress *lrgAB*. These experiments revealed that impaired aerobic growth of the Δ*lrgAB* mutant can be effectively restored by supplementation of pyruvate, and that perturbated expression of *lrgAB* significantly affects pyruvate flux and the conversion of pyruvate to acetyl-CoA by the Pdh pathway, verifying that LrgAB is closely linked to pyruvate catabolism. *In vitro* competition assays revealed that LrgAB plays an important role in *S. mutans* competition with H_2_O_2_-producing *S. gordonii*, an interaction which can also be modulated by external pyruvate. However, no obvious competitive disadvantage was observed against *S. gordonii* by either the *S. mutans lrgAB* mutant or *lrgAB* overexpression strain *in vivo* using a mouse caries model. Organic acid analysis of mouse dental biofilms revealed that metabolites produced by the host and/or dental plaque microbiota could complement the deficiency of a *lrgAB* mutant, and favored *S. mutans* establishment compared to *S. gordonii*. Collectively, these results reinforce the importance of the oral microbiota and the metabolic environment in the oral cavity battleground, and highlight that pyruvate uptake through LrgAB may be crucial for interspecies competition that drives niche occupancy.

## Introduction

Cariogenic bacteria such as *Streptococcus mutans* are very effective at metabolizing a wide range of carbon sources, producing strong organic acids, and surviving in acidic environments, collectively allowing these organisms to thrive in the oral cavity of humans consuming a variety of diets (Burne, 1998; Marsh, 2003; Burne et al., 2009). In particular, the ability of *S. mutans* to efficiently acquire carbohydrates and coordinate its metabolism in response to environmental fluctuations is critical for outcompeting non-cariogenic indigenous oral microbiota in caries-conducive conditions. These abilities are also closely linked to the capacity of *S. mutans* to withstand a variety of stressors encountered in the oral cavity. All these processes are complex and cross-regulated with multiple metabolic pathways and virulence properties (Lemos and Burne, 2008; Smith and Spatafora, 2012).

Over the past decade, we have studied the *S. mutans* LrgAB system, originally hypothesized to induce cell death and lysis with its partner operon CidAB, and subsequently shown to play a significant role in modulating a variety of key *S. mutans* virulence traits, including autolysis, biofilm formation, oxidative and heat stresses, and genetic competence (Ahn et al., 2010, 2012, 2017; Rice et al., 2017). Interestingly, the *lrgAB* mutation was also shown to affect major metabolic pathways associated with carbohydrate, amino acid, fatty acid/lipid, nucleotide metabolism and transport (Ahn et al., 2017; Rice et al., 2017). Recently, we reported that LrgAB functions as a stationary phase pyruvate uptake system (Ahn et al., 2019) and its function is regulated by two global regulators, CcpA (Kim et al., 2019) and CodY (Ahn et al., 2020c), that are important for cellular responses to altered carbohydrate availability and amino acid limitations (Henkin, 1996; Sonenshein, 2005, 2007; Deutscher, 2008; Gorke and Stulke, 2008). More interestingly, we demonstrated that excess pyruvate produced during growth can be subsequently utilized as a carbon source during stationary phase (Ahn et al., 2019, 2020a), presumably promoting long-term survival of *S. mutans* when in a metabolically competitive environment with other oral microbiota. Given that pyruvate is a central carbon metabolite directly linked to key metabolic pathways and has a relatively fast-turnover during stationary phase, environmental pyruvate (or other metabolites) may therefore modulate interspecies competition during caries development. Although the role and regulation of LrgAB have been relatively well characterized, its ecological importance has not yet been fully investigated. In this study, we characterize how LrgAB contributes to the competitive fitness of *S. mutans in vitro* and *in vivo*, using a dual-species model with *S. gordonii*, a H_2_O_2_-producing oral commensal. The experimental outcomes described herein provide guidance for further defining the cariological and ecological significance of LrgAB.

## Materials and Methods

### Growth Conditions

All *S. mutans* and *S. gordonii* strains were grown in BHI (brain heart infusion) medium (Difco) as overnight cultures at 37°C in a 5% CO_2_ atmosphere. When necessary, antibiotics were added to cultures as follows: kanamycin (1 mg/ml) and spectinomycin (1 mg/ml). Overnight cultures were diluted 1:50 into fresh BHI broth, grown to mid-exponential phase (OD_600_∼0.4–0.5), and then used as seed cultures for the assays described below. The media used for these assays included BHI, chemically defined FMC (Terleckyj and Shockman, 1975; Terleckyj et al., 1975), and TV (Tryptone/Vitamin) (Burne et al., 1999), the latter two supplemented with 11 mM glucose (named FMC11 and TV11, respectively), as BHI already contains 11 mM glucose. Each medium was supplemented with α-ketoglutaric acid (αKG; Sigma-Aldrich), oxaloacetic acid (OA; Sigma-Aldrich), or sodium pyruvate (pyr; Fisher Scientific), at concentrations as indicated for each experiment. To achieve anaerobic conditions, sterile mineral oil was placed on top of the cultures.

### Bacterial Strains and Mutant Construction

*S. mutans* strains used in this study were UA159 (wildtype) and isogenic *lrgAB*-deficient mutant (Δ*lrgAB*) (Ahn et al., 2010), *lrgAB*-overexpression strain (SAB161), and *lytST*-overexpression strain (SAB163) (Ishkov et al., 2020). The SAB161 strain, constitutively expressing *lrgAB*, was constructed as previously described (Ahn and Rice, 2016). Briefly, the promoter region of *lrgA* (P*lrgA*) was replaced by a fragment (ΩKm-P*ldh*) containing a polar kanamycin resistance gene (ΩKm) and a *ldh* promoter region (P*ldh*). For this, two ∼0.5 kb fragments flanking the −35 and −10 sequences of the *lrgA* promoter were PCR-amplified, ligated into the ΩKm-P*ldh* cassette, previously generated by PCR amplification (Ahn and Rice, 2016), and transformed into *S. mutans*. Transformants were selected on BHI agar containing kanamycin, and double-crossover recombination was confirmed by PCR and sequencing. A GFP reporter assay to monitor P*lrgA* activity consisted of the previously created P*lrgA-gfp* construct (Kim et al., 2019) in the shuttle vector pDL278 (LeBlanc et al., 1992), transformed into SAB161. As a control strain, the empty pDL278 plasmid was also transformed in this mutant. The GFP reporter and control constructs in wildtype, Δ*lrgAB*, and SAB163 backgrounds were previously generated (Kim et al., 2019; Ahn et al., 2020b; Ishkov et al., 2020). For a dual-species competition assay with *S. mutans*, *S. gordonii* wildtype DL1 (generating H_2_O_2_ in a SpxB-dependent manner) and Δ*spxB* (*spxB*-deficient) strains (Huang et al., 2016) were used.

### Microplate Assays

To monitor growth patterns over time, seed cultures of each strain were diluted 1:100 into 350 μl fresh medium in individual wells of a 100-well honeycomb plate and the optical density at 600 nm (OD_600_) measured at 37°C at 30 min intervals using a Bioscreen C growth curve analysis system. At least three independent experiments, each in quadruplicate, were performed. A representative result is presented in each relevant figure. To monitor *lrgAB* expression (P*lrgA* activity) over growth, *S. mutans* strains harboring a P*lrgA-gfp* reporter fusion or pDL278 were used, as described above. Seed cultures of each reporter strain were diluted 1:50 into 175 μl fresh medium in individual wells of a 96-well plate (black walls, clear bottoms; Corning), which was subsequently loaded into a Synergy microplate reader (BioTek) controlled by Gen5 software (Ahn et al., 2019, 2020a; Kim et al., 2019). The optical density for growth curves and green fluorescence intensity for *lrgAB* expression were monitored at 600 and 485 nm/520 nm (excitation/emission), respectively, at 30 min intervals for 18–24 h. The fluorescence of wildtype harboring the plasmid without the reporter gene fusion was subtracted from fluorescence readings of each *S. mutans* strain harboring the P*lrgA-gfp* gene fusion. At least three independent replicates, each in triplicate, were performed. A representative result is presented in each relevant figure.

### Measurement of Extracellular Pyruvate Levels

*S. mutans* UA159 wildtype, SAB161, and SAB163 were grown in FMC medium containing 11 mM glucose. Measurements of extracellular pyruvate during growth were taken from 250 μl samples at 1–2 h intervals and 100 μl was used to monitor growth (OD_600_). Extracellular pyruvate levels during early-exponential growth and stationary phase were predicted based on previously published data (Ahn et al., 2019). Thus, pyruvate quantification in this study was focused on samples taken at late-exponential and early-stationary phases, time points at which *lrgAB* induction and LrgAB-dependent pyruvate uptake occur. The remaining 150 μl of each sample was centrifuged for 2 min at 18,000 × g to remove cells, and pyruvate concentrations of supernatants quantified with an EnzyChrom^TM^ pyruvate assay kit (BioAssay Systems, Hayward, CA, United States), according to the manufacturer’s instructions.

### Quantitative Real-Time PCR (qPCR) Assay

To measure growth-dependent gene expression of *lrgA*, *lytS*, *pdhC*, and *pfl*, using qPCR, *S. mutans* wild-type and isogenic mutant strains were grown in BHI, at 37°C in a 5% (vol/vol) CO_2_ atmosphere, and cells were harvested at early-exponential (EE; OD_600_∼0.2) and -stationary (ES; OD_600_∼1.0) phase. RNA extraction, reverse-transcriptase (RT) reaction, qPCR, and data analysis were performed as described elsewhere (Ahn et al., 2012; Ahn and Rice, 2016; Rice et al., 2017). Expression values were normalized against an internal standard (*gyrA*) and presented as relative copy number of mRNA (copies/μl) for comparison with each gene. Statistical analyses were performed on data generated from *n* = 3 independent experiments using an unpaired *t*-test.

### *In vitro* Competition Assays

For dual-species competition assays between each *S. mutans* strain (UA159, Δ*lrgAB*, SAB161, or SAB163) and *S. gordonii* strain (DL1 or Δ*spxB*), 200 μl of each competing seed culture (one from *S. mutans* and the other from *S. gordonii*), were each adjusted to OD_600_ = 0.5 in BHI medium, centrifuged, the pellet washed with PBS (phosphate buffer saline), resuspended in 200 μl PBS, and then diluted 1:100 into 200 μl fresh medium in individual wells of a 96-well plate (Costar^TM^3595, Corning). After 24 h incubation at 37°C in an aerobic atmosphere, 5 μl culture from each well was inoculated on Prussian blue agar plates (Saito et al., 2007), indicating the production of H_2_O_2_. The plates were incubated for an additional 24 h at 37°C in an aerobic atmosphere. Growth and blue precipitation zones were documented and analyzed with VisionWorks^®^ software (Analytik Jena, Upland, CA, United States). Original grayscale .tif images of each plate were each converted to RGB color, and mid-tone color balanced (−50, 0, +31) in Adobe Photoshop 21.2.1 to improve the contrast and clarity of each image.

### Murine *in vivo* Caries Model

A mouse caries protocol was performed as previously described (Culp et al., 2015; Shields et al., 2018) with modifications. All procedures with solutions and samples were performed under BSL2 conditions and mice were kept under ABSL2 conditions. Briefly, inbred 3-weeks-old female SPF BALB/cJ mice (The Jackson Laboratory, Bar Harbor, ME, United States) were placed in pairs in sterile cages and grouped into four groups of 20 mice. Two days later, mice were given drinking water containing 0.8 mg/ml sulphamethoxazole/0.16 mg/ml trimethoprim for a total of 10 days to suppress indigenous oral bacteria, followed by a 3-day washout period with sterile drinking water. On the following day (designated experimental day 0) mice were placed on a modification of powdered AIN-93G purified diet containing 37.5% (wt/vol) total sucrose (Cat. # TD.160810, Envigo, Madison, WI, United States). Mice were also given 4% (wt/vol) sterile sucrose water. In initial colonization experiments, mice were inoculated on experimental day 0 and each successive 4 days with 50 μl of 1.5% (wt/vol) carboxymethylcellulose containing approximately 1 × 10^9^ cells of either wild type *S. mutans* UA159 (WT), Δ*lrgAB* (*lrgAB*-deficient), SAB161 (*lrgAB*-overexpressing) or with carboxymethylcellulose alone (mock inoculations). In competition experiments, mice were first inoculated over five consecutive days starting on experimental day 0 with 50 μl 1.5% (wt/vol) carboxymethylcellulose containing approximately 1 × 10^9^ cells of wild type *S. gordonii* DL1 followed on experimental day 7 with five consecutive daily inoculations with 50 μl 1.5% (wt/vol) carboxymethylcellulose containing approximately 1 × 10^9^ cells of either wild type *S. mutans* UA159 (WT), Δ*lrgAB* (*lrgAB*-deficient), SAB161 (*lrgAB*-overexpressing) or with carboxymethylcellulose alone (mock *S. mutans*). Oral swabs were taken at intervals to monitor colonization using HydraFlock^®^ 6” Sterile Micro Ultrafine Flock swabs (Puritan Medical Products, Guilford, ME). At the end of each experiment mice were euthanized by CO_2_ asphyxiation followed by cervical dislocation. The protocol was reviewed and approved by the Institutional Animal Care and Use Committee at University of Florida (IACUC protocol #201810470).

Swab tips were vortexed (3 times for 5 s) in 1 ml sterile PBS, the tips removed and 200 μl added of ice-cold PBS containing approximately 5 × 10^8^ depurinated cells of laboratory strain *S. mitis* UF2. The tube was then vortexed 5 s and centrifuged (10,000 × *g*, 10 min at 4°C) to pellet recovered cells. Cell pellets were then processed for DNA isolation using the DNeasy UltraClean Microbial kit (Qiagen Inc., Germantown, MD, United States) as per manufacturer’s instructions. In preliminary experiments, employment of a high concentration of depurinated cells was found to greatly enhance quantitative pelleting and recovery of low cell numbers and subsequent DNA, thus increasing the sensitivity of qPCR assays. The cells walls of depurinated cells remain intact and therefore at high concentrations likely act as a carrier to help limit non-specific binding and promote pelleting of recovered bacteria. Purine bases in genomic DNA are lost by depurination, producing apurinic sites and rendering DNA undetectable in all three qPCR assays. Cells from a 200 ml culture (OD_600_ = 0.5) of laboratory strain *S. mitis* UF2 in BHI were pelleted (4 × 50 ml at 10,000g × 7 min at 4°C) and each pellet resuspended in 11 ml sterile ice-cold PBS. Cell were pooled and again centrifuged. The pellet was resuspended in 35 ml of 0.2 N HCl and placed in a 70°C water bath for 90 min with vortexing (5 × 2 s) every 15 min. Cells were then pelleted as before and the 90 min incubation in fresh 0.2 N HCl repeated. Cells were washed 3 times with 30 ml sterile ice-cold PBS. Before the third centrifugation the cell concentration was estimated from OD_600_ and the subsequent cell pellet resuspended in sterile ice-cold PBS to a concentration of approximately 2.5 × 10^9^ cells/ml, then aliquoted and stored at −75°C.

To assess dental colonization, the left and right halves of each mandible were aseptically extracted by first breaking the fibrous symphysis at the rostral midline, then gripping one incisor and pulling the left or right half of mandibular bone away from the temporal mandibular joint and nearly all associated soft tissue. Then, under a dissecting microscope, any remaining extraneous soft tissue near the molar teeth was removed by scraping with a scalpel followed by removal of bone approximately 2 mm anterior and posterior to the three molar teeth. Molar teeth with remaining underlying bone were sonicated on ice in 1 ml sterile PBS, pH 7.4, in siliconized 2 mL microcentrifuge tubes. Molar teeth with remaining bone were then aseptically removed using sterile forceps. Approximately 5 × 10^8^ depurinated cells of laboratory strain *S. mitis* UF2 were then added, the tube vortexed 5 s and centrifuged (10,000 × *g*, 10 min at 4°C) to pellet recovered cells. Cell pellets were then processed for DNA isolation as described above for swabs. Supernatants were then immediately used for analyses of organic acids as described below.

Quantitative PCR was used to estimate total recovered bacterial genomes and recovered genomes of inoculated strains in each DNA sample. DNA isolation using the DNeasy UltraClean Microbial kit (Qiagen) resulted in 50 μl of DNA that was diluted to 125 μl with nuclease free water resulting in DNA in 4 mM Tris-HCl, pH 8.0. Samples from swabs were stored at −75°C in aliquots. Samples from molars were treated similarly, but further diluted 10-fold with 4 mM Tris-HCl, pH 8.0 to eliminate interference in qPCR assays caused by unknown components in the samples, then aliquoted and stored at −75°C. Each qPCR assay included 9 μl of diluted DNA and resultant genome numbers from the average of triplicates then multiplied by either 13.89 (for swab DNAs) or 138.89 (for mandibular DNAs) to calculated total recovered genomes in each sample. To estimate total recovered bacteria, degenerate primers were used to PCR amplify conserved regions of the ubiquitous single-copy gene, *rpsL* (30S ribosomal protein S12) (Lang et al., 2013; Shields et al., 2018). Recovery of mouse commensals was then estimated by subtracting recovered genomes for inoculated strains from total recovered bacterial genomes. Primers and qPCR conditions used are given in Supplementary Table S1. Standard curves were derived from DNA samples isolated from each strain grown to mid-exponential phase in BHI. *S. mutans* UA159 was used as standard for *rpsL* assays. Efficiencies, slopes and *r*^2^ values for standard curves were greater than 90%, −3.205 and 0.978, respectively. Statistical comparisons of colonization between groups were by one-way ANOVA with Tukey’s multiple comparisons test after conversion of genomes recovered to their log values in order to stabilize variances.

Smooth surface and sulcal caries of mandibular and maxillary molars were scored by a single calibrated examiner using Larson’s modification of the Keyes’ scoring system, as described previously (Culp et al., 2015). The linear evaluations of carious enamel involvement are expressed as E, while severities of carious lesions, based on degree of dentin involvement, are expressed as Ds (dentin exposed) and Dm (3/4 of the dentin affected). To stabilize variances, caries scores were expressed as proportions of their maximum possible values (124 for smooth surface caries and 56 for sulcal surface caries) and then the arcsine of the square root of the proportions calculated, as described previously (Culp et al., 2005). Transformed scores were compared by analysis of variance with the Tukey-Kramer *post hoc* test using Prism v8.1 (GraphPad Software, San Diego, CA, United States).

### Organic Acid Analysis of Mouse Plaque Samples

The supernatants from sonicates of mandibles from four mice were pooled, yielding five samples of pooled supernatants for each group of twenty mice. Each pooled supernatant was then aspirated into a sterile 5 ml Luer-Lok^TM^ syringe using a 16-gauge needle (BD). The needle was removed and a 0.2 μm, 13 mm PVDF disposable filter was fastened to the 5 mL syringe (GE Whatman). Each sample was then filtered into a sterile 15 ml tube (Corning). Any sample remaining in the filter was collected by passing 5 ml of air through the filter. Samples were centrifuged at 1,000 × g for 1 min at 4°C to collect supernatants to the bottom of each 15 ml tube. The tubes were then frozen at −75°C and lyophilized. Metabolomics experiments and analyses were performed in the Metabolomics Core of CVI (Cardiovascular Institute) at University of Pennsylvania. Briefly, each lyophilized sample was dissolved in 150 μl of 50% MeOH and vortexed for 3 min. Thereafter, centrifugation was carried out at 10,000 rpm for 10 min, and 100 μl of the supernatant was obtained. The resuspended metabolite samples (10 μl) were injected onto Luna NH2 column (Phenomenex, CA, United States). LC-MS/MS analysis was performed using Agilent 6420 triple quadrupole (Agilent, CA, United States). Data Acquisition software was used to monitor and control LC-MS. Metabolites were separated on the analytical column at a flow rate of 150 μl/min and the LC gradient method was set as follows: *t* = 0 min, 85% B; *t* = 15 min, 0% B; *t* = 38 min, 0% B; *t* = 40 min, 85% B; *t* = 50 min, 85%. Solvent A was 20 mM ammonium acetate + 20 mM ammonium hydroxide in 95:5 water: acetonitrile and solvent B was 100% acetonitrile. The separated metabolites were identified by dynamic multiple reaction monitoring (MRM). Dynamic MRM involves sorting for the retention time of a specific parent molecular ion, the parent molecular weight, the optimized collision energy to produce a specific fragment ion and the fragment ion. Before sample analysis, authentic samples of carboxylic acids were used to verify dynamic MRM transition for quantitative dynamic MRM analysis. Using dynamic MRM, metabolites were identified, and relative quantification was determined by area of the peak in the chromatogram of each metabolite. Data was normalized by protein quantification.

## Results

### Either Lack of Expression or Overexpression of *lrgAB* Causes Induction of *lrgAB* Expression at Stationary Phase

**FIGURE 1:**
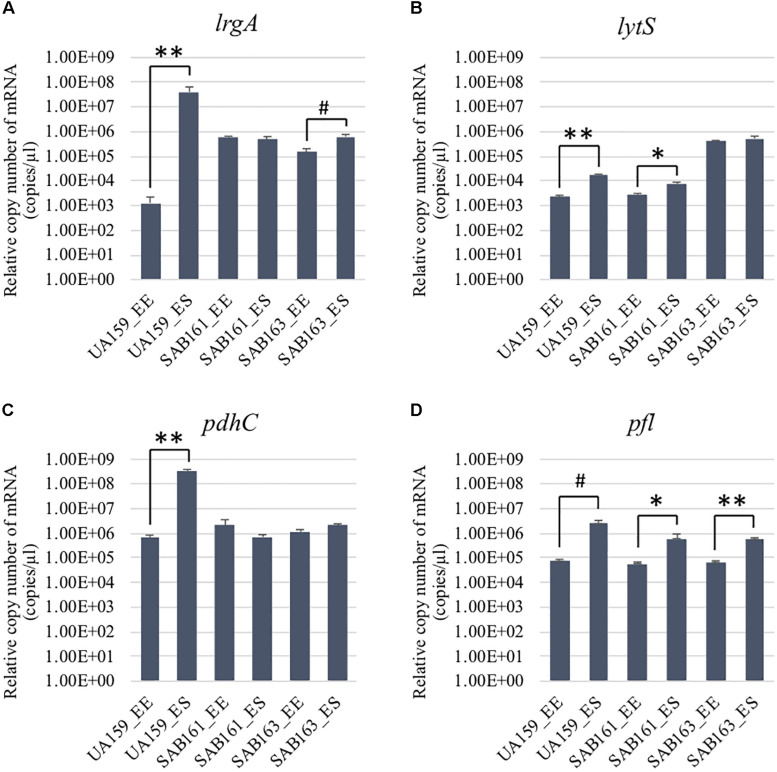
Growth-dependent expression determined by qRT-PCR in the *lrg* derivative strains. The expression of *lrgA* **(A)**, *lytS*, **(B)** *pdhC* **(C)**, and *pfl* **(D)** was measured at early-exponential (EE; OD_600_∼0.15–0.2) and -stationary (ES; OD_600_∼1.0) growth phases of *S. mutans* UA159 (WT), SAB161 (*lrgAB*-overexpressing), and SAB163 (*lytST*-overexpressing) (see section “Materials and Methods” for details). Data are averages of three independent biological replicates. Differences in relative gene expression between strains were evaluated for statistical significance by Student’s *t-*test (unpaired; one-tailed). **P < 0.001; *P < 0.005; ^#^*P* < 0.01.

To further characterize the role and regulation of LrgAB, a *lrgAB* constitutive overexpression strain of *S. mutans* UA159 was created (SAB161), whereby *lrgAB* expression was placed under control of the *S. mutans ldh* promoter and ribosome binding site (RBS). For comparison, we also included SAB163 (Ahn et al., 2020a), recently created to overexpress *lytST* in a similar manner to SAB161. Given that expression of *lrgAB* is under tight positive regulation by LytST (Ahn et al., 2010, 2012), *lrgAB* is also overexpressed in SAB163, which was recently observed by GFP quantification assays using a P*lrgA-gfp* reporter strain in SAB163 (Ahn et al., 2020a). We compared expression of *lrgAB* and *lytST* transcripts in wildtype (UA159), SAB161 and SAB163 at early-exponential (EE) and early-stationary (ES) growth phases by qPCR (Figure 1). We first confirmed that *lrgA* was dramatically upregulated at ES (> about 4-logs), compared to EE in the wild-type strain, while such a remarkable stationary-phase induction was not observed during the transition from EE to ES in either SAB161 or SAB163 (Figure 1A), although a slight increase was observed at ES in SAB163. This observation confirmed that *lrgAB* expression is constitutively over-expressed in SAB161 and is independent of growth phase. However, SAB163 still maintained a modest induction of *lrgAB* expression in stationary phase, and overexpressed *lytST* was responsible for increased *lrgAB* expression at EE (relative to wildtype), as recently observed (Ahn et al., 2020a). Consistent with our previous observation (Ahn et al., 2012), *lytS* expression levels in the wildtype were approximately 7.7-fold elevated during the transition to stationary phase (Figure 1B). This increment was moderately alleviated in SAB161, showing only a 2.6-fold increase, suggesting that *lytST* may be subject to possible feedback regulation though the *lytST-lrgAB* circuit. As expected, *lytS* was overexpressed in SAB163 throughout growth and its expression was at least a log higher than that observed in wildtype and SAB161 (Figure 1B). Thus, these two derivative strains, SAB161 and SAB163, overexpressing *lrgAB* during growth, were further investigated in this study, in conjunction with a Δ*lrgAB* mutant strain (referred to below as “*lrgAB* deficient”).

We recently reported that stationary phase P*lrgA* activation was further increased in both the Δ*lrgAB* and SAB163 strains, relative to wildtype (Ahn et al., 2020a, b; Ishkov et al., 2020). For measurement of P*lrgA* activity in SAB161 during growth, the P*lrgA-gfp* construct was introduced into SAB161 and P*lrgA* activity compared between the wildtype, Δ*lrgAB*, SAB161 and SAB163 backgrounds. Interestingly, P*lrgA* activity was increased about threefold in SAB161 at stationary growth (Supplementary Figure S1C), compared to that in wildtype (Supplementary Figure S1A). This induction was approximately 2.5-fold higher than in the Δ*lrgAB* background (Supplementary Figure S1B), and about 40% lower than in the SAB163 background (Supplementary Figure S1D). Another interesting finding was that the uncontrolled overexpression of *lrgAB* (in SAB 161) had no effect on P*lrgA* activity during exponential growth, confirming that P*lrgA* is specifically activated upon entry into stationary phase. This is in line with the sharp induction of high-level P*lrgA* activity in SAB163 between late-exponential and stationary growth (Supplementary Figure S1D), consistent with our previously published results (Ahn et al., 2020a). However, overexpression of *lytST* (SAB163) remarkably relieved the repression of *lrgAB* during exponential growth (Supplementary Figure S1D). Therefore, these observations demonstrate that either lack of expression or overexpression of *lrgAB* triggers the ability of *S. mutans* to induce *lrgAB* expression at stationary phase, although these effects could be exerted through different cellular and metabolic routes.

### Either Lack of Expression or Overexpression of *lrgAB* Promotes Pyruvate Excretion During Growth

**FIGURE 2:**
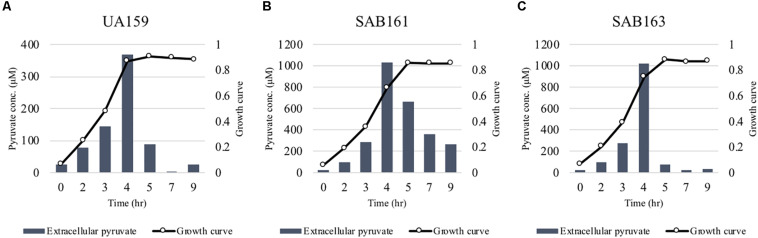
Measurement of extracellular pyruvate during growth of the *lrg* derivative strains. The strains, including *S. mutans* UA159 (WT, **A**), SAB161 (*lrgAB*-overexpressing, **B**), and SAB163 (*lytST*-overexpressing, **C**) were cultivated in FMC medium supplemented by 11 mM glucose. For time course measurements of extracellular pyruvate and growth, samples were taken at 1 or 2 h intervals (see section “Materials and Methods” for details). The concentration of pyruvate was determined using an EnzyChrom^TM^ pyruvate assay kit, and growth was measured by the optical density at 600 nm (OD_600_). Bars indicates the average concentration of extracellular pyruvate; solid line with circles indicates the corresponding growth curve. The results are representative of two independent experiments.

**FIGURE 3:**
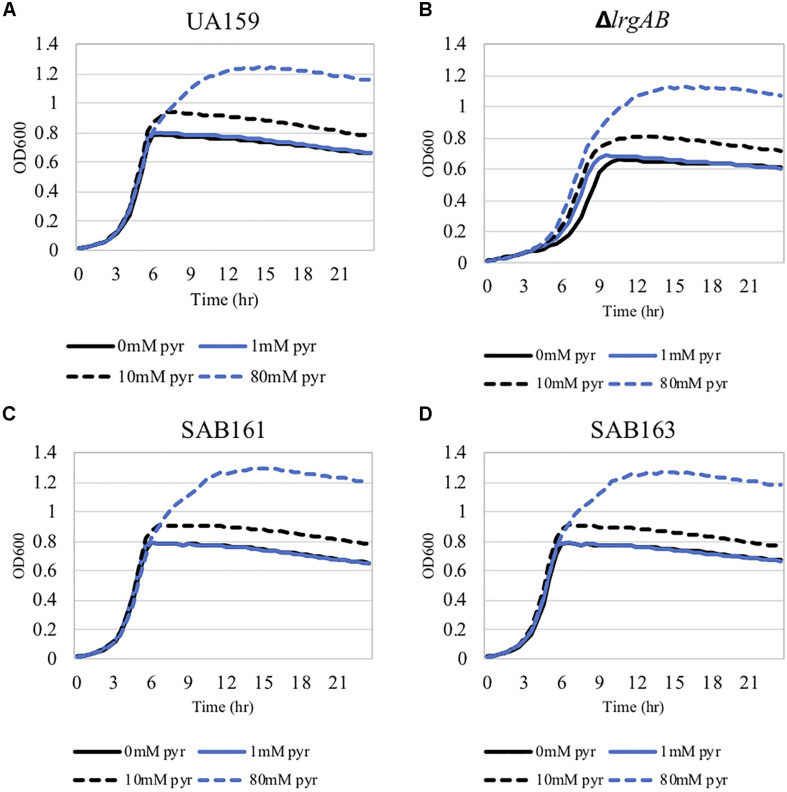
The effect of exogenously added pyruvate on the aerobic growth of the *lrg-*derivative strains. The strains, including *S. mutans* UA159 (WT, **A**), Δ*lrgAB* (*lrgAB*-deficient, **B**), SAB161 (*lrgAB*-overexpressing, **C**), and SAB163 (*lytST*-overexpressing, **D**) in BHI medium supplemented by different concentrations of exogenous pyruvate (0, 1, 10, and 40 mM). OD_600_ was monitored every 30 min at 37°C using the Bioscreen C lab system. The results are representative of three independent experiments.

We previously demonstrated that the maximum level of pyruvate excreted during growth of the Δ*lrgAB* strain was about 50% elevated compared to wildtype (Ahn et al., 2019), implying that stationary phase *lrgAB* induction may be positively correlated with excreted pyruvate levels. Thus, we cultivated wildtype, SAB161 and SAB163 under the same conditions used previously to assess extracellular pyruvate levels during growth (FMC11 medium, at 37°C in a 5% CO_2_ atmosphere) (Ahn et al., 2019). As shown Figures 2B,C, the maximum level (∼1,000 μM) of pyruvate excreted during growth of the SAB161 and SAB163 strains was about threefold higher than wildtype (∼350 μM, Figure 2A), which may contribute to the observed increase in P*lrgA* activity observed in these strains (Supplementary Figures S1C,D). Stationary phase pyruvate re-uptake in SAB161 was somewhat decelerated (Figure 2B) relative to the wildtype (Figure 2A) and SAB163 (Figure 2C) strains. These results suggest that lack of expression or overexpression of *lrgAB* can each increase pyruvate excretion compared to wildtype *S. mutans*, although its underlying mechanism remains unknown. Thus, we next evaluated how either lack of expression or overexpression of *lrgAB* impacts the capacity of this organism to respond to and take up external pyruvate, particularly during aerobic growth, as oxygen availability was shown previously to influence the efficacy of LrgAB to function as a pyruvate uptake system (Ahn et al., 2019), consequently contributing to phenotypes associated with *lrgAB* expression. We aerobically cultivated *S. mutans* wildtype and derivative strains in BHI, supplemented by different concentrations (0, 1, 10, and 40 mM) of exogenous pyruvate. All strains efficiently utilized external pyruvate to sustain growth at stationary phase (Figure 3), in accordance with increased *lrgAB* promoter activity at stationary phase (Supplementary Figure S1). As expected, the Δ*lrgAB* strain experienced delayed exponential growth and entered stationary phase at a lower overall OD_600_ (Ahn et al., 2010; Ahn and Rice, 2016) relative to wildtype (Figures 3A,B). However, the impaired aerobic growth phenotype of the Δ*lrgAB* mutant was considerably restored by addition of either 10 mM or 80 mM pyruvate (Figure 3B). This growth restoration effect was not previously recognized when the cell was tested in FMC medium supplemented by pyruvate (Ahn et al., 2019), suggesting that the effect of LrgAB deficiency may depend on specific external metabolic/nutritional conditions. It is also noteworthy that exogenous pyruvate promoted growth of all strains to a similar extent, despite considerably different stationary phase P*lrgA* activities (Supplementary Figure S1), suggesting that the LrgAB-mediated pyruvate uptake and its cellular consequence are not proportional to the level of *lrgAB* expression, possibly due to post-transcriptional effects or metabolic adjustments.

### Lack or Overexpression of *lrgAB* Alleviates the Conversion of Pyruvate to Acetyl-CoA

**FIGURE 4:**
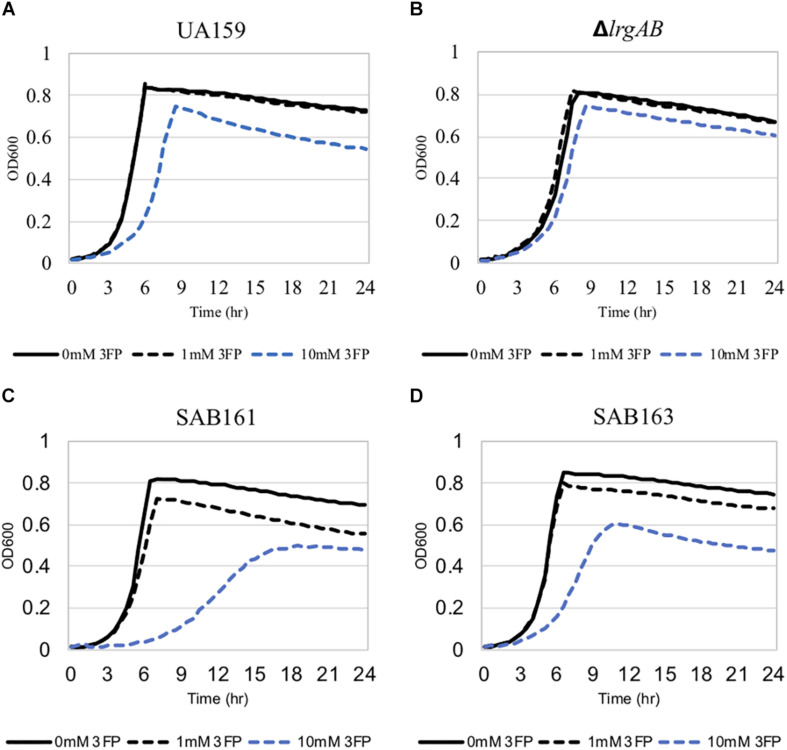
The effect of 3-fluoropyruvate (3FP; pyruvate analog) on the growth of the *lrg-*derivative strains. The strains, including *S. mutans* UA159 (WT, **A**), Δ*lrgAB* (*lrgAB*-deficient, **B**), SAB161 (*lrgAB*-overexpressing, **C**), and SAB163 (*lytST*-overexpressing, **D**), were cultivated in BHI medium supplemented by different concentrations of 3FP (0, 1, and 10 mM). Optical density at 600 nm was monitored every 30 min at 37°C using the Bioscreen C lab system. The results are representative of three independent experiments.

The finding that exogenous pyruvate restores the impaired aerobic growth phenotype of the Δ*lrgAB* strain (Figure 3B), further supports our previous hypothesis that external pyruvate may be internalized into the cell during exponential growth, independently of LrgAB (Ahn et al., 2019). We reported previously that stationary phase *lrgAB* induction and pyruvate uptake are efficiently inhibited by low concentrations (∼0.01 mM) of the toxic pyruvate analog, 3FP (3-fluoropyruvate) (Ahn et al., 2019). This analog competes with pyruvate and inhibits cell growth by binding to cellular pyruvate dehydrogenase (Pdh) complexes (Lang et al., 1987; Flournoy and Frey, 1989). *S. mutans* was unable to normally grow in the presence of 10 mM 3FP (Ahn et al., 2019). In this study, when we cultivated *S. mutans* wildtype and derivative strains in BHI, containing different levels (0, 1, and 10 mM) of 3FP, the Δ*lrgAB* strain was more resistant to 3FP than wildtype, particularly in the presence of 10 mM 3FP (Figure 4B). In contrast, SAB161 and SAB163 displayed elevated sensitivity even at low (1 mM) concentrations of 3FP (Figures 4C,D, respectively), compared to the wildtype and Δ*lrgAB* strains (Figures 4A,B, respectively), and their growth was even more severely impaired in the presence of 10 mM 3FP (Figures 4C,D) compared to wildtype. When this experiment was repeated in TV medium, a growth condition in which induction of *lrgAB* is not observed (Ahn et al., 2020a), growth trends in the presence of 3FP were similar to those observed in BHI, except that growth impairments were more severe in all strains tested (Supplementary Figure S2). Therefore, these results suggest that external pyruvate may be taken up by the cell through LrgAB during growth, which may subsequently affect metabolic enzymes such as Pdh. To investigate whether either lack of expression or overexpression of *lrgAB* actually affects expression of pyruvate metabolic pathways, we used qPCR to measure growth-dependent expression of *pdhC* transcripts, encoding one of the components of the Pdh complex, in wildtype, SAB161 and SAB163 strains. As shown in Figure 1C, the *pdhC* transcript levels were about 500-fold elevated during the transition from EE (early-exponential) to ES (early-stationary) in the wildtype, which is consistent with our previous microarray data (Ahn et al., 2012, 2019). However, this induction was completely abolished in both the SAB161 and SAB163 strains (Figure 1C). Given that the Pdh enzymes are responsible for the conversion of pyruvate to acetyl-CoA, this result suggests that pyruvate utilization via Pdh may be interrupted when *lrgAB* is overexpressed. In the previous study with the Δ*lytS* mutant all *pdhDABC* genes were still considerably upregulated at ES (approximately 45–212-fold) compared to EE, although upregulation was smaller than in the wildtype (approximately 284–366-fold). Collectively, these results suggest that overexpression of *lrgAB*, and to a lesser extent the absence of *lrgAB*, interferes with the catabolism of pyruvate through the Pdh pathway. Pyruvate is also converted by pyruvate formate lyase (Pfl) to acetyl-CoA and formate, particularly in the absence of oxygen. Interestingly, expression of *pfl* was increased about 32-fold as wildtype cells approached stationary phase when grown in a 95% air – 5% CO_2_ atmosphere (Figure 1D). This expression pattern is comparable to the approximately 38-fold increase in wildtype *pfl* expression observed in our previous microarray experiment (Ahn et al., 2012). The magnitude of *pfl* induction was less alleviated in SAB161 and SAB163, approximately 10- and 8.5-fold, respectively (Figure 1D). In our previous microarray experiment, expression of *pfl* in the Δ*lytS* strain was still about 35-fold increased during the transition to stationary phase (Ahn et al., 2012). Although the Pfl enzyme is unlikely to be active in this culture condition (5% CO_2_), it is possible that regulation of *pfl* gene expression is affected by both lack of expression and overexpression of *lrgAB*. These data also suggest that LrgAB, responsible for stationary phase pyruvate uptake, is closely linked to pyruvate catabolism.

### Contribution of LrgAB to Competition Between *S. mutans* and *S. gordonii*, *in vitro*

**FIGURE 5:**
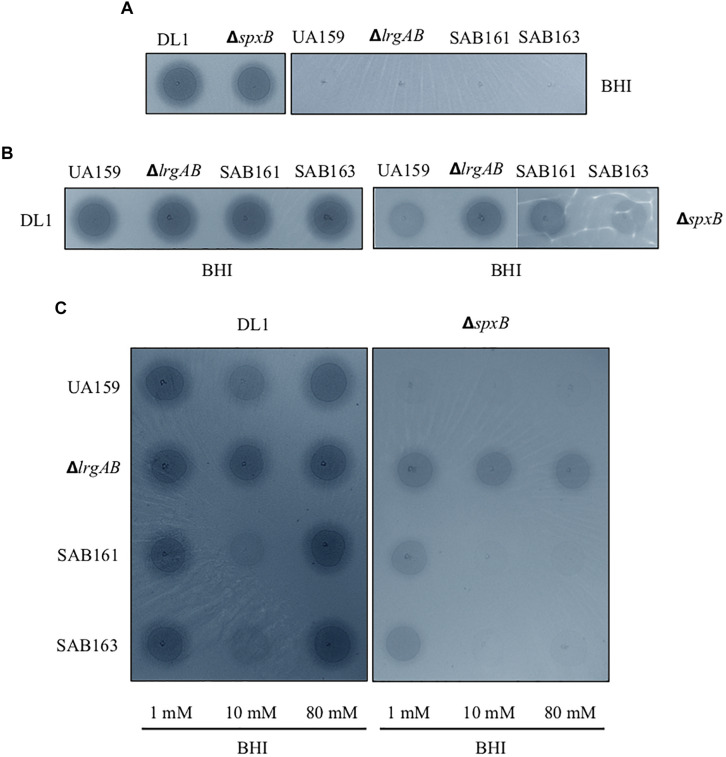
Dual-species competition assays between *S. mutans lrg*-derivative strains and *S. gordonii* DL1, or Δ*spxB*. The *lrg-*derivative strains include *S. mutans* UA159 (WT), Δ*lrgAB* (*lrgAB*-deficient), SAB161 (*lrgAB*-overexpressing), and SAB163 (*lytST*-overexpressing). Each *S. mutans* strains was cultured singly **(A)** or together in competition with either *S. gordonii* DL1 (left panels of B and C) or Δ*spxB* (right panels of **B,C**) in plain BHI medium **(A,B)** or in BHI medium supplemented by 1, 10, and 80 mM exogenous pyruvate **(C)**. All cultures were for 24 h at 37°C in an aerobic atmosphere, and then was inoculated on Prussian blue agar plates. After an additional 24 h, the growth and blue precipitation zone were observed and analyzed with VisionWorks^®^ software. All images were subjected to identical color balancing settings in Adobe Photoshop as described in section “Materials and Methods.”

Given the fast-turnover nature of pyruvate upon nutrient limitation, the re-uptake of pyruvate through LrgAB may represent a competitive advantage for *S. mutans* against other endogenous species of the oral microbiome. To this end, we further explored how LrgAB contributes to *S. mutans* competition with other oral streptococci. We utilized a dual-species competition model, *in vitro*, with H_2_O_2_-generating *S. gordonii*, because LrgAB has been previously shown to be especially important for coping with oxidative stress, such as H_2_O_2_ challenge (Rice et al., 2017). Each *S. mutans* derivative strain and *S. gordonii* wildtype DL1 was cultured on Prussian blue agar medium, widely used to detect the presence of H_2_O_2_ (Saito et al., 2007). As shown in Figure 5A, *S. gordonii* DL1 produced a blue precipitation zone indicating H_2_O_2_ production, while *S. mutans* produced no distinct precipitation zone. Thus, when *S. mutans* cells outcompete *S. gordonii* cells, the blue precipitation zone should be reduced. However, the *S. gordonii* strain Δ*spxB*, lacking SpxB (pyruvate oxidase) that mediates production of H_2_O_2_ in the presence of oxygen, also produced a blue precipitation zone which was slightly smaller than wildtype DL1 yet still distinct from *S. mutans* (Figure 5A), suggesting that Δ*spxB* is still capable of producing H_2_O_2_ or another oxidant that reacts with this stain (presumably by an SpxB-independent mechanism). When each *S. mutans* strain was co-cultured with wildtype DL1 for 24 h and spotted onto Prussian blue agar medium, the sizes of blue zones among *S. mutans* strains were not qualitatively different (Figure 5B, left panel) and were also similar to DL1 alone as seen in Figure 5A. Thus, *S. gordonii* DL1 outcompeted all *S. mutans* strains. In contrast, when co-cultured with Δ*spxB*, all *S. mutans* strains, except for Δ*lrgAB*, appeared to be more competitive against Δ*spxB*, as manifested by generation of less blue precipitate by the *spxB* mutant (Figure 5B, right panel). These results suggest that LrgAB contributes to the ability of *S. mutans* to cope with H_2_O_2_ challenge, but this contribution is overshadowed by the higher levels of H_2_O_2_ generated by *S. gordonii* wildtype DL1.

To investigate whether external pyruvate influences the interaction between *S. mutans* and *S. gordonii*, we added pyruvate at concentrations of 1, 10, and 80 mM to the co-cultures of *S. mutans* and *S. gordonii* strains. Supplementation with 1 mM pyruvate resulted in no obvious difference in competition between any of the *S. mutans* strains and DL1 (Figure 5C, leftmost column of left panel). However, in the presence of 10 mM pyruvate, *S. mutans* SAB161 and SB163 were more competitive against DL1 (Figure 5C, middle column of left panel), indicating LrgAB in the presence of high concentrations of external pyruvate can function to enhance the competitiveness of *S. mutans* against *S. gordonii*. Interestingly, this competitive advantage for *S. mutans* growth was not observed in the presence of 80 mM pyruvate, due possibly to negative feedback regulation for stationary phase *lrgAB* induction and pyruvate uptake in response to high concentrations of external pyruvate (≥40 mM) (Ahn et al., 2019). In competition with Δ*spxB*, a much clearer competitive advantage for *S. mutans* strains was observed at all concentration of pyruvate, except for Δ*lrgAB* (Figure 5C, right panel), further suggesting LrgAB in the presence of external pyruvate confers a competitive fitness to *S. mutans* when coping with oxidative stress.

### Contribution of LrgAB to *S. mutans* Competition With *S. gordonii, in vivo*

To interrogate whether LrgAB mediates a competitive advantage for *S. mutans* against *S. gordonii in vivo*, as well as to evaluate the cariological potential of *S. mutans* when LrgAB is absent or overexpressed, we used a well-defined mouse caries model (Culp et al., 2005, 2011). First, we used qPCR assays to compare oral colonization (estimated from oral swabs at days 10, 29, and 48, following the initial inoculation, Supplementary Figure S3A) and dental colonization (from sonicates of mandibular molars) by each of the three *S. mutans* strains (wildtype, Δ*lrgAB* or SAB161) plus a fourth uninoculated group as a negative control (mock). In addition, colonization by the total indigenous mouse commensal bacteria was assessed (Supplementary Figure S3B). All three *S. mutans* strains colonized the oral cavity at similar levels, gradually displaying an increase at day 48 versus day 10. Dental colonization by all three strains were also similar (Supplementary Figure S3B). Interestingly, recovery of mouse commensal bacteria increased with time when mice were inoculated with either of the *S. mutans* strains, whereas in the absence of *S. mutans* (mock group), the number of recovered commensal bacteria was unchanged. However, recovered commensals at day 10 in all three *S. mutans* groups were significantly less than in the mock group, indicating all *S. mutans* strains had a negative initial impact on oral commensal bacteria. However, as a whole, the commensal bacterial community was able to eventually grow. Recoveries of *S. mutans* strains and mouse commensal bacteria from molar biofilms were, in all cases, not statistically different, although the average values were consistently higher for each *S. mutans* strain compared to commensals. Caries scores reflected this colonization status (Supplementary Table S2), showing that the absence or overexpression of *lrgAB* had no obvious effect on the cariogenicity of *S. mutans* in this model.

**FIGURE 6:**
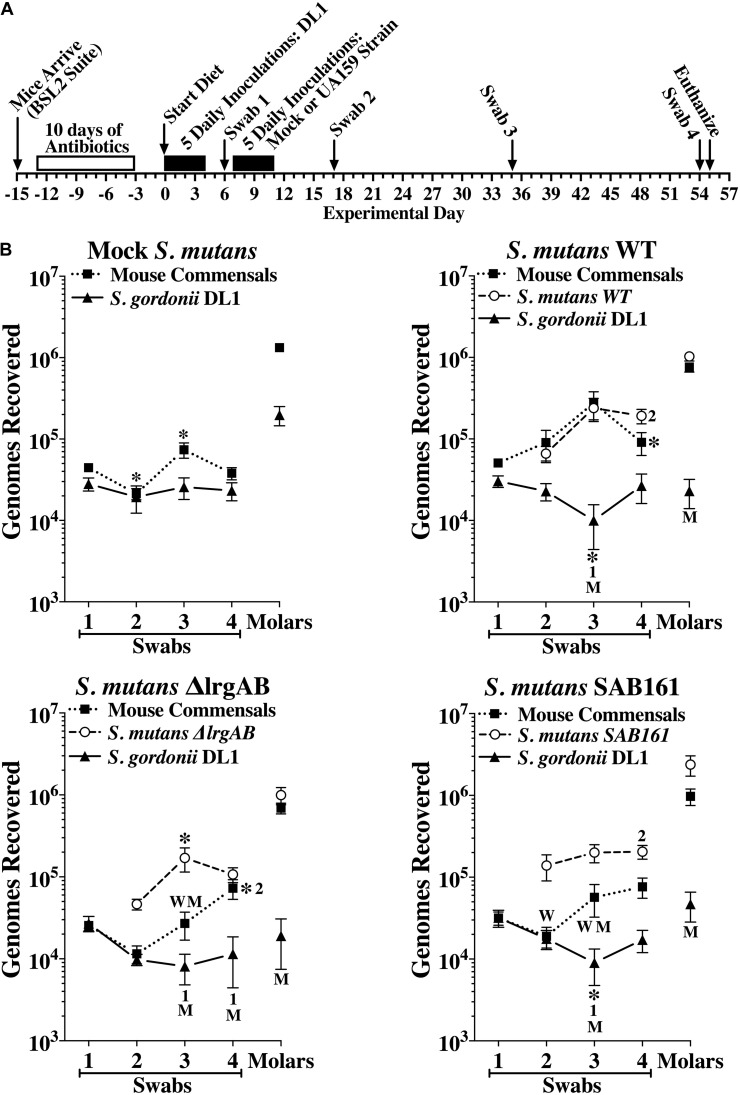
Colonization of the oral cavity and mandibular molars of mice by wild type *S. mutans* UA159 (WT), Δ*lrgAB* (*lrgAB*-deficient) and SAB161 (*lrgAB*-overexpressing) strains with *S. gordonii* DL1. **(A)** Timeline of key events in the experiment (see section “Materials and Methods” for details). **(B)** Colonization results for each indicated inoculated strain and mouse oral commensals (Commensals) from oral swabs 1–4 and from sonicates of mandibular molars taken at experimental days indicated in the timeline. Results are mean ± SE (*n* = 20 mice per group) of recovered genomes estimated by qPCR. Statistical comparisons by one-way ANOVA with Tukey-Kramer multiple comparisons test. *p ≤ 0.05 versus the previous swab or an earlier swab as indicated by the swab number, or versus the same point in either the mock (M) or wild type (W) group.

In a second *in vivo* experiment, we further examined the contribution of LrgAB to competitiveness of *S. mutans* against *S. gordonii* DL1. The experimental design is depicted in Figure 6A. All mice were first inoculated with a single pure culture of *S. gordonii* DL1. One week later, three groups of 20 mice were inoculated with either *S. mutans* wildtype, Δ*lrgAB* or SAB161, while the fourth group (Mock *S. mutans*) underwent inoculations without added *S. mutans*. Oral colonization by *S. gordonii* DL1 was consistent over time in the mock group, whereas oral colonization by DL1 in the presence of all three strains of *S. mutans* was negatively impacted at day 35, but were able to recover to levels seen in the mock group by day 54, except in the Δ*lrgAB* group, in which DL1 levels remained below the mock group (Figure 6B). The establishment of DL1 on molars was hindered by subsequent inoculation with all of the *S. mutans* strains (Figure 6B), which is in contrast to the *in vitro* data, in which *S. gordonii* DL1 was dominant against *S. mutans* on Prussian Blue agar medium plates (Figure 5B, left panel). Both oral colonization and incorporation of *S. mutans* into dental biofilms were similar between all three strains of *S. mutans*, although oral colonization, but not dental colonization, appeared lower in the presence of DL1 (Figure 6B) versus in its absence (Supplementary Figure S3B). With respect to mouse commensal bacteria, DL1 had no apparent impact on either oral or dental colonization (compare upper left panels in Figure 6B with Supplementary Figure S3B). Recoveries of mouse commensals from oral swabs after challenge with each of the three strains of *S. mutans* showed some minor variations but remained mostly persistent. The presence within the dental biofilm of mouse commensals was unimpeded when challenged with either of these three *S. mutans* strains. Again, caries scores reflected colonization status (Supplementary Table S3), suggesting that either lack or overexpression of *lrgAB* had no major impact on competition with *S. gordonii.*

### Deficiency of *lrgAB* May Be Complemented by Other External Metabolites

**FIGURE 7:**
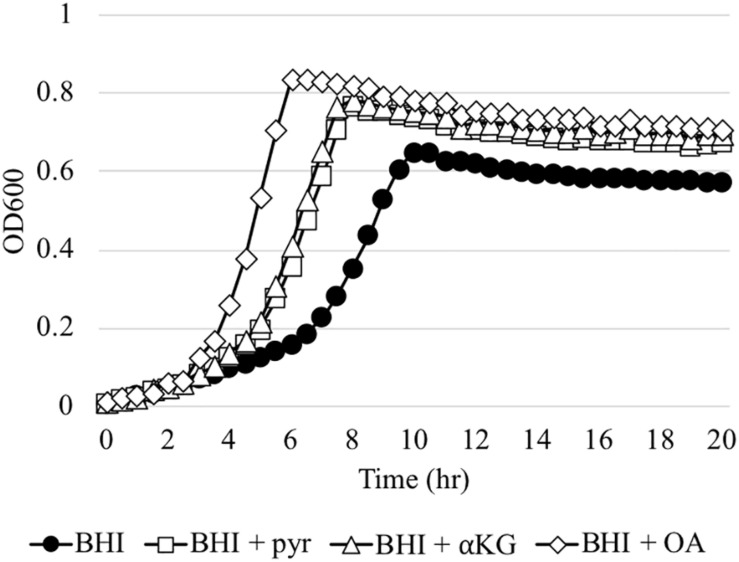
The effect of α-keto-acids on the aerobic growth impairment of the Δ*lrgAB* mutant strain. The Δ*lrgAB* strain was aerobically cultivated in plain BHI medium and BHI supplemented by 1 mM exogenous pyruvate (pyr), α-ketoglutarate (αKG), and oxaloacetate (OA). Optical density at 600 nm was monitored every 30 min at 37°C using the Bioscreen C lab system. The results are representative of three independent experiments.

The *in vivo* competition data above was somewhat surprising, because even the Δ*lrgAB* strain displayed successful colonization of molar biofilms, in which the surrounding micro-environment is likely aerobic. In fact, when the Δ*lrgAB* strain is aerobically cultured on an agar plate, growth of the mutant is almost completely inhibited (Ahn et al., 2010). However, as shown above in Figure 3B, the impaired aerobic growth phenotype of the Δ*lrgAB* mutant was restored *in vitro* by external pyruvate (Figure 3B). *S. mutans* and other members of the oral microbiota can excrete various metabolic organic acids, particularly α-keto acids, including pyruvate (pyr), α-ketoglutarate (αKG) and oxaloacetate (OA), which are known to possess H_2_O_2_-scavenging activity (Nath et al., 1995; Giandomenico et al., 1997; Long and Halliwell, 2011, 2012). Both αKG and OA restored *S. mutans*’ growth in the presence of a H_2_O_2_ challenge (Supplementary Figure S4), similar to pyruvate (Ahn et al., 2019). However, unlike pyruvate, αKG and OA did not promote enhanced growth at stationary phase when challenged with H_2_O_2_, suggesting that these organic acids may not be utilized as a secondary carbon source in this growth condition. However, αKG and to a great extent OA restored the aerobic growth defect of the Δ*lrgAB* mutant strain (Figure 7). These observations suggest that common metabolites, such as α-keto acids, within the oral microflora may have impacted the outcomes of our *in vivo S. gordonii* DL1 – *S. mutans* competition experiment. Efforts were thus directed to analyze samples from this experiment by mass spectrometry for a profile of organic acids in supernatants after pelleting cells from mandibular sonicates, an acellular extract of molar biofilms. Results between each of the four groups of mice are shown in Supplementary Table S4 and demonstrate remarkably high concentrations of lactate in all four groups, likely due to carbohydrate fermentation by oral resident bacteria, even in the absence of *S. mutans* (mock group), suggesting that a high proportion of acidogenic and aciduric species could already reside in the mouse oral microbiota. Pyruvate concentrations in all groups were relatively low compared to most other organic acids, possibly due to its fast-turnover. Interestingly, there were no statistically significant differences between groups for each of the assayed organic acids, suggesting extracellular metabolites may be dynamically balanced by microbiota within dental biofilms in response to ecological fluctuations, even within the more acidic environment imparted by *S. mutans*.

## Discussion

In this present study, we further characterize the role and regulation of LrgAB in *S. mutans* focusing on its cariological and ecological significance to better understand its function in a condition mimicking the *in vivo* oral milieu. One important finding is that LrgAB is closely linked to the conversion of pyruvate to acetyl-CoA through the Pdh pathway, likely modulating both the intracellular and overflow (excreted) levels of pyruvate. Although the underlying mechanisms remain to be elucidated, overexpression of *lrgAB* almost completely inhibits induction of the *pdh* genes in *S. mutans* that are normally dramatically upregulated during the transition to stationary growth phase (Ahn et al., 2012). Lack of *lrgAB*, which observed in the Δ*lytS* mutant, also appeared to modestly alleviate stationary phase *pdh* induction, relative to that observed in the wildtype. Thus, metabolic changes in response to perturbated expression of *lrgAB* may have a negative effect on the activity of Pdh. In line with this supposition, either lack or overexpression of *lrgAB* promoted pyruvate excretion during *S. mutans* growth. Intimate linkage between LrgAB and pyruvate catabolism was anticipated from our previous results (Ahn et al., 2020b) in which stationary-phase *lrgAB* induction and pyruvate uptake were also completely inhibited by disruption of the Pta-AckA pathway, responsible for the conversion of acetyl-CoA to acetate with the production of one molecule of ATP. Collectively, these findings suggest the catabolic rate of pyruvate is an important trigger to take up external pyruvate through LrgAB, and *vice versa*. Results, moreover, are consistent with previous results demonstrating both *lrgAB* and *pta-ackA* expression are directly under the control of two global regulators CcpA and CodY (Kim and Burne, 2017; Kim et al., 2019; Ahn et al., 2020c). Considering Pdh is highly regulated by various metabolic intermediates, including pyruvate (Hansen and Henning, 1966; Schwartz and Reed, 1970; Shen and Atkinson, 1970; Datta, 1991), and that *pdh* genes are commonly upregulated in response to three environmental stressors (i.e., aerobic, heat and vancomycin challenge) (Rice et al., 2017), it is reasonable to posit that pyruvate catabolism and *lrg* regulation work together and are modulated by the cell’s metabolic status and environmental condition.

Another notable finding is that *lrgAB* expression was correlated with the magnitude of growth inhibition by 3FP, known to interfere with the cellular Pdh complex (Lang et al., 1987; Flournoy and Frey, 1989), thus suggesting external pyruvate is taken up through LrgAB during exponential growth, in addition to the stationary phase (Ahn et al., 2019). This possibility was also suggested by our recent observation, showing that the addition of pyruvate to tryptone medium elicited P*lrgA* activation during exponential growth and an enhanced growth rate (Ahn et al., 2020a). It therefore is plausible that pyruvate is dynamically fluxed through LrgAB in response to external pyruvate levels, or other unknown metabolic/nutritional signals, whereas uptake and utilization of external pyruvate for re-growth may require activation of the LytST-LrgAB circuit by a metabolic cue.

Deciphering the competitive fitness between species within the oral microbiota is crucial for understanding the contribution of LrgAB to the pathogenic potential of *S. mutans* and changes that lead to dysbiosis. This present study therefore employed a dual-species model of *S. mutans* and *S. gordonii* to further characterize the ecological significance of LrgAB. In competition assays using Prussian blue agar medium, we found *S. gordonii* DL1 was relatively dominant over all tested *S. mutans* derivative strains when cultured aerobically, although this was not surprising given that DL1 generates H_2_O_2_ under ample oxygen in a SpxB-dependent manner (Kreth et al., 2005, 2008). However, LrgAB contribution to this competition was not evident. As anticipated, DL1 dominance was attenuated in an isogenic *spxB*-deficient mutant (Δ*spxB*), which only displayed dominance against the Δ*lrgAB* strain, reinforcing a role for LrgAB in *S. mutans’* competition with H_2_O_2_-generating *S. gordonii*. Given that pyruvate is excreted as an overflow metabolite, it should be a common carbon source in microbiome environments such as the oral cavity (Takahashi et al., 2010; Gawron et al., 2019). More interestingly, external pyruvate modulated this interspecies competition, with 10 mM pyruvate conferring a great competitive advantage to *S. mutans* wildtype and SAB161 but not Δ*lrgAB.* This advantage may be due to the ability of pyruvate to scavenge H_2_O_2_, as evidenced by excretion of more pyruvate by *S. mutans* during growth in the presence of 10 mM pyruvate than in its absence, enabling more efficient and rapid scavenging of H_2_O_2_. Also, pyruvate at 10 mM may be more efficiently utilized by *S. mutans* compared to DL1 to promote its growth at stationary phase. The loss of this advantage by increasing exogenous pyruvate to 80 mM may be due to negative feedback regulation through LytST, as observed by ≥40 mM pyruvate (Ahn et al., 2019). Conversely, 80 mM pyruvate may have enhanced DL1’s capacity to generate H_2_O_2_, as its dominance was lost upon deletion of *spxB.* Although the molecular events behind these observations cannot be fully clarified under the present experimental approach, these results may have implications for therapeutic application of pyruvate to restrict the survival of *S. mutans* or enhance the fitness of H_2_O_2_-producing non-cariogenic bacteria.

The outcomes of competition between *S. mutans* and DL1 *in vivo* were rather unexpected, in that both the absence or overexpression of *S. mutans lrgAB* had little effect on competitiveness against DL1. Moreover, despite prior dental colonization by DL1, *S. mutans* consistently outnumbered DL1 leading to high caries scores in all experimental groups. Considering the Δ*lrgAB* mutant is highly sensitive to oxygen and H_2_O_2_ (Ahn et al., 2010; Turner et al., 2019), and given the superiority of DL1 in competition with all three strains of *S. mutans in vitro*, our *in vivo* results indicate this dual-species competition is affected considerably by *in vivo* environmental factors. Such factors include interactions with other oral microbiota, host factors and diet. The highly cariogenic mouse diet may have imparted a competitive advantage for *S. mutans*, although Tanzer et al. (2012) previously reported that *S. mutans’* competitiveness against DL1 is independent of a high sucrose diet. In addition, in another *in vivo* study, we found strains of human commensal streptococci that are competitive with *S. mutans* under the same high sucrose diet (manuscript submitted). Another possibility is that the micro-environment within the dental biofilm is micro-aerobic, therefore better supporting growth of the Δ*lrgAB* mutant compared to a more aerobic condition. Dietary sucrose triggers production of extracellular glucans by *S. mutans*, creating an anaerobic microenvironment (Kleinberg, 2002; Paes Leme et al., 2006; Bowen and Koo, 2011; Koo et al., 2013) that may be disadvantageous to DL1, as it requires oxygen to generate competitive amounts of H_2_O_2_. This anoxic biofilm environment may also complement the aerobic growth impairment of the Δ*lrgAB* strain. *S. mutans* also utilizes dietary sucrose to produce acid, thus lowering the surrounding pH and leading to a shift in the oral microbiota composition toward a higher proportion of other acidogenic and aciduric species (Marsh, 2003; Takahashi and Nyvad, 2008; Bradshaw and Lynch, 2013; Rosier et al., 2014). The fact that a comparable amount of lactate was detected even in the mock group of mice not inoculated with *S. mutans*, suggests select mouse commensal species within dental biofilms also produce lactic acid during fermentation of sucrose, that may help foster *S. mutans’* colonization. Moreover, the extremely low levels of caries in the mock group further suggests acids produced by mouse commensals are likely diffused away from tooth enamel surfaces, possibly due to significantly less production of a surrounding extracellular glucan-like matrix. Because total recovered mouse commensals from dental biofilms were similar between the mock group and the three *S. mutans* groups, one or more species of mouse commensals are likely also aciduric and thus flourish even when in competition with *S. mutans*. Future *in vivo* studies of dual-species competition may consider the use of gnotobiotic mice, a less extreme cariogenic diet or delineation of colonization by specific indigenous species and their phenotypes.

The observation that DL1 failed to compete with the oxygen-sensitive *S. mutans* Δ*lrgAB* strain *in vivo*, extended our interest in the ecological role of external metabolites in interspecies competition, which was initiated by our finding that external pyruvate restored impaired aerobic growth of the Δ*lrgAB* strain. Subsequently, we found the same effect when supplementing the culture with other α-keto acids, such as α-ketoglutarate and oxaloacetate, further supporting an ecological contribution of metabolic intermediates excreted presumably by indigenous microbiota. The ability of α-keto acids to buffer external sources of oxidative stress, such as H_2_O_2_, may have assisted the oxygen-sensitive Δ*lrgAB* strain to cope with oxidative stress *in vivo*. The reaction of α-keto acids, such as α-ketoglutarate, oxaloacetate, and pyruvate, with H_2_O_2_ is expected to generate CO_2_ and either acetate, succinate or malonate as major byproducts (Giandomenico et al., 1997; Oh et al., 2002; Berry and Toms, 2006; Satpute et al., 2014). Acetate, in particular, would also be taken up into the cell in parallel with pyruvate under nutrient-limited growth conditions (Jolkver et al., 2009; Paczia et al., 2012), and was recently reported to affect stationary phase *lrgAB* induction in *S. mutans* (Ahn et al., 2020a). Changes in levels of α-keto acids was also reported to coordinate catabolism of amino acids and glucose in *Escherichia coli* (Zampieri et al., 2019). These dynamically changing external metabolites could force bacteria to continuously modulate their uptake, which in turn may require continuous redirection of intracellular fluxes. These external metabolites may therefore have modulated the resources available for indigenous microbiota, and also affected the capacity of DL1 to produce competitive amounts of H_2_O_2_. Mouse host factors (e.g., salivary peroxidase) could also attenuate localized H_2_O_2_ concentrations (Tenovuo, 1976; Tenovuo and Pruitt, 1984; Carlsson, 1987; Ericson and Bratt, 1987). In fact, H_2_O_2_ itself is unstable and readily degraded in nature. These factors combined may have attenuated establishment of DL1 within the indigenous microbiota community.

We also compared organic acids from molar sonicates to explore correlations between the metabolic activity of oral microbiota in dental biofilms and the contribution of LrgAB to caries development. Surprisingly, the abundance of each organic acid was similar among each of the four groups, suggesting the exo-metabolome may be rapidly balanced through continuous adjustments of intercellular fluxes and cross-competition within the murine oral indigenous microbiota. Nevertheless, it is noteworthy that together with glucose, malate is the preferred carbon source for *B. subtilis* and is known to repress transcription of *pftAB* (previously, *ysbAB*), a *lrgAB* homolog in this organism (Charbonnier et al., 2017). Our previous transcriptomic analysis also showed that the *mleS* gene (SMU.137), encoding an enzyme catalyzing conversion of L-malate to lactic acid and CO_2_, was > 3-fold downregulated in the Δ*lrgAB* strain compared to the wildtype (Rice et al., 2017). This malolactic enzyme was further proposed to enhance the acid tolerance response by increasing the cytoplasmic pH through production of CO_2_ (Sheng and Marquis, 2007). However, given caries and recovered malate levels were similar between the wildtype and Δ*lrgAB* strains, the Δ*lrgAB* strain must use alternative and/or redundant mechanisms for acid tolerance, in addition to metabolism of malate. Nevertheless, it may be interesting to study how malate uptake and metabolism are linked to *lrg* and pyruvate uptake regulation.

In summary, the present study implemented the use of both *in vitro* and *in vivo* competition models to address how LrgAB contributes to niche competition and modulates the cariogenic potential of *S. mutans*. The results suggest that the role and regulation of LrgAB are coordinated in response to both external and internal metabolic fluxes and dynamics that are more variable *in vivo*, and consequently precluded detection of a specific cariological and ecological role for LrgAB. Although knowledge of niche competition with non-mutans streptococci in rodents with indigenous oral flora is limited, this *in vivo* study opens the possibility of examining the relationship between ecological fitness and metabolite fluxes that has been overlooked in interpreting the outcomes of *in vivo* interspecies competition experiments.

## Data Availability Statement

All datasets presented in this study are included in the article/Supplementary Material.

## Ethics Statement

The animal study was reviewed and approved by the Institutional Animal Care and Use Committee at University of Florida.

## Author Contributions

S-JA contributed to conception, design, acquisition, analysis, and interpretation, and drafted the manuscript. WH and SD contributed to acquisition, analysis, and interpretation. KR contributed to conception and interpretation, and edited the manuscript. DC contributed to conception, design, acquisition, analysis, and interpretation, and edited the manuscript. All authors gave the final approval and agreed to be accountable for all aspects of the work.

## Conflict of Interest

The authors declare that the research was conducted in the absence of any commercial or financial relationships that could be construed as a potential conflict of interest.
