# Supplementary material for: Understanding LrgAB Regulation of Streptococcus mutans Metabolism
Source: Front Microbiol. 2020 Sep 3;11:2119. doi: 10.3389/fmicb.2020.02119 (PMC7496758; doi:10.3389/fmicb.2020.02119)
Supplement: Supplementary file 1 [file Data_Sheet_1.PDF]

Supplementary Material

Table S1. qPCR primers and reaction conditions.

Total bacteria	
Target locus:	*rpsL*, 30S ribosomal subunit protein S12
Forward primer:	5'-CCKAAYTCNGCNYTNCGTAA-3'
Reverse primer:	5'-CGHACMCCWGGWARGTCYTT-3'
Annealing:	54°C
Elongation:	60°C
Amplicon length:	149 bp
S. mutans UA159	Reference Sequence: NC_004350.2
Target locus:	locus_tag SMU.292
Forward primer:	5'-TGGCAAGTCCTGATGGTTTGAC-3'
Reverse primer:	5'-GGAAGCGGAAGCTGTGATGAAC-3'
Annealing/elongation:	70°C
Amplicon length:	142 bp
S. gordonii DL1	Reference Sequence: NC_009785.1
Target locus:	locus_tag SGO_RS08055
Forward primer:	5'-CGCACGATAAATTGACAGA-3'
Reverse primer:	5'-CGCCATTCATCATACCATAA-3'
Annealing/elongation:	67°C
Amplicon length:	162 bp

Reactions of 20 µl were run in triplicate in a Bio-Rad CFX96 real-time PCR instrument using 10 µl SsoAdvanced Universal SYBR®Green Supermix (Bio-Rad, Hercules, CA), 0.5 µl each primer and 9 µl DNA. Samples were run at 3 min at 98°C followed by either 34 cycles for rpsL (98°C, 15 s; annealing temperature as indicated, 20 s; elongation temperature as indicated, 20 s; followed by a melt curve at 65-95°C at 0.5°C increments) or 40 cycles (98°C, 15 s; annealing/elongation temperature as indicated, 45 s; followed by a melt curve at 65-95°C at 0.5°C increments). All primers were used at 0.50 µM final concentration except for *rpsL* (1.0 µM). Results were analyzed using the Bio-Rad CFX Manager program. Under the listed conditions, assays for *S. gordonii* DL1 and *S. mutans* UA159 failed to amplify each other's DNA or mouse oral commensal DNA isolated from oral swabs taken prior to administrating antibiotic water. All work was performed in a BioSafety cabinet under aseptic conditions.

Table S2. Sulcal and smooth surface caries and severity on molar teeth, plus smooth surface caries development by surface: Comparisons between BALB/cJ mice inoculated with *S. mutans* UA159 (wildtype, WT), the indicated derivative strain or administered mock inoculations with carrier only.

SMOOTH SURFACES	Mock	WT	Δ*lrgAB*	SAB161
**Total - E**	0.55 (0.17)	15.95 (1.91)†	16.75 (1.74)†	17.20 (2.50)†
**Total - Ds**	0.00 (0.00)	8.10 (1.37)†	6.35 (1.02)†	7.85 (1.48)†
**Total - Dm**	0.00 (0.00)	2.85 (0.51)†	1.65 (0.20)†	3.70 (0.74)†
**Buccal - E**	0.45 (0.17)	7.65 (1.03)†	7.15 (0.93)†	7.25 (1.27)†
**Buccal - Ds**	0.00 (0.00)	3.60 (0.74)†	2.35 (0.56)†	3.30 (0.78)†
**Buccal - Dm**	0.00 (0.00)	1.05 (0.39)†	0.30 (0.16)†	1.20 (0.36)†
**Lingual - E**	0.05 (0.05)	7.65 (0.75)†	9.20 (0.78)†	8.75 (1.01)†
**Lingual - Ds**	0.00 (0.00)	4.10 (0.50)†	3.95 (0.48)†	3.80 (0.55)†
**Lingual - Dm**	0.00 (0.00)	1.70 (0.15)†	1.35 (0.17)†	2.05 (0.29)†
**Proximal - E**	0.05 (0.05)	0.65 (0.32)	0.40 (0.20)	1.20 (0.38)*
**Proximal - Ds**	0.00 (0.00)	0.40 (0.23)	0.05 (0.05)	0.75 (0.31)*
**Proximal - Dm**	0.00 (0.00)	0.10 (0.10)	0.00 (0.00)	0.45 (0.24)*
**SULCAL SURFACES**				
**Total - E**	0.55 (0.17)	23.70 (1.63)†	24.50 (1.62)†	24.55 (2.05)†
**Total - Ds**	0.00 (0.00)	16.95 (1.84)†	18.90 (1.77)†	17.10 (2.15)†
**Total - Dm**	0.00 (0.00)	3.45 (0.76)†	3.15 (0.86)†	3.45 (0.78)†

Values are means (SE) of Larson's modified Keyes' scores (N = 20 each group). Total smooth surface caries is sum of buccal, lingual and proximal caries. E (enamel affected), Ds (dentin exposed) and Dm (3/4 of the dentin affected). Caries scores were compared using ANOVA with the Tukey-Kramer post hoc test. †, $p < 0.0001$ versus Mock; *, $p < 0.05$ versus Mock. In all cases, $p > 0.05$ in comparisons within each score category between groups inoculated with a UA159 strain, with the exception of proximal Dm UA159 ΔSAB161 versus UA159 ΔlrgAB, $p < 0.05$.

Table S3. Development of caries and their severities on molars comparing mice inoculated with *S. gordonii* DL1 mock inoculations (DL1) versus mice inoculated with *S. gordonii* DL1 followed one week later with either *S. mutans* UA159 WT (DL1 + WT), *S. mutans* SAB161 (DL1 + SAB161) or *S. mutans* Δ*lrgAB* (DL1 + Δ*lrgAB*).

**SMOOTH SURFACES**	**DL1**	**DL1 + WT**	**DL1 + SAB161**	**DL1 + Δ*lrgAB***
**Total - E**	1.10 (0.22)	18.05 (2.03)*	19.80 (2.17)*	19.00 (1.97)*
**Total - Ds**	0.00 (0.00)	5.55 (0.96)†	6.35 (0.89)*	4.35 (1.12)‡
**Total - Dm**	0.00 (0.00)	1.50 (0.34)γ	2.60 (0.48)*	1.30 (0.33)γb
**Total - Dx**	0.00 (0.00)	0.05 (0.05)	0.25 (0.14)	0.00 (0.00)
**Buccal E**	0.02 (0.09)	7.85 (1.10)*	8.05 (0.94)*	7.45 (0.84)*
**Buccal Ds**	0.00 (0.00)	2.25 (0.58)‡	2.30 (0.48)‡	1.00 (0.48)
**Buccal Dm**	0.00 (0.00)	0.40 (0.22)	0.70 (0.19)γ	0.25 (0.16)
**Buccal Dx**	0.00 (0.00)	0.05 (0.05)	0.05 (0.05)	0.00 (0.00)
**Lingual E**	0.70 (0.16)	8.70 (0.77)*	10.05 (0.97)*	9.60 (0.90)*
**Lingual Ds**	0.00 (0.00)	3.05 (0.36)*	3.60 (0.40)*	3.05 (0.55)*
**Lingual Dm**	0.00 (0.00)	1.00 (0.19)‡	1.70 (0.30)*	1.05 (0.23)‡
**Lingual Dx**	0.00 (0.00)	0.00 (0.00)	0.20 (0.12)	0.00 (0.00)
**Proximal E**	0.20 (0.09)	1.50 (0.34)*	1.70 (0.47)γ	2.25 (0.40)†
**Proximal Ds**	0.00 (0.00)	0.25 (0.20)	0.45 (0.21)	0.30 (0.18)
**Proximal Dm**	0.00 (0.00)	0.10 (0.10)	0.20 (0.14)	0.00 (0.00)
**Proximal Dx**	0.00 (0.00)	0.00 (0.00)	0.00 (0.00)	0.00 (0.00)
**SULCAL SURFACES**				
**Total - E**	4.60 (0.64)	23.65 (1.40)*	28.50 (1.15)*c	21.05 (1.13)*a
**Total - Ds**	0.05 (0.05)	8.70 (1.37)*	8.55 (0.98)*	6.40 (0.93)*
**Total - Dm**	0.00 (0.00)	3.85 (0.80)*	3.75 (0.66)*	1.85 (0.35)
**Total - Dx**	0.00 (0.00)	0.00 (0.00)	0.00 (0.00)	0.00 (0.00)

Values are means (SE) of Larson's modified Keyes' scores. 20 mice per group. Total smooth surface caries is sum of buccal, lingual and proximal caries. E (enamel affected), Ds (dentin exposed), Dm (3/4 of the dentin affected), and Dx (whole dentin affected). Comparisons by ANOVA with Tukey Honest Significant Difference post-test. *****, $P < 0.0001$ versus DL1; †, $P < 0.0002$ versus DL1; ‡, $P < 0.002$ versus DL1; γ, $P < 0.033$ versus DL1; a, $P < 0.0001$ versus DL1 + SAB161; b, $P < 0.033$ versus DL1 + SAB161; c, $P < 0.033$ versus DL1 + UA159.

Table S4. Analysis by mass spectrometry of organic acids in acellular extracts from sonicates of mandibular molars.

	Organic Acid (nmol/mg protein)							
**Group**	**3HBA**	**α-Ketoglutarate**	**Citrate**	**Fumarate**	**Lactate**	**Malate**	**Pyruvate**	**Succinate**
**Mock *S. mutans***	3.77<br>(1.73)	1.36<br>(0.43)	43.24<br>(13.28)	14.63<br>(8.77)	1,767.89<br>(787.13)	82.13<br>(46.89)	8.54<br>(1.78)	4.50<br>(1.70)
**WT**	3.73<br>(0.57)	1.06<br>(0.34)	35.44<br>(4.26)	13.48<br>(3.67)	1,664.63<br>(272.49)	72.27<br>(24.01)	7.07<br>(2.35)	3.35<br>(1.44)
ΔlrgAB	5.02<br>(1.55)	1.65<br>(0.76)	53.23<br>(21.48)	20.42<br>(6.33)	1,803.26<br>(479.41)	101.54<br>(11.98)	8.99<br>(3.99)	4.09<br>(1.56)
**SAB161**	4.57<br>(1.20)	1.64<br>(0.51)	49.59<br>(10.29)	19.51<br>(9.13)	1,801.44<br>(194.51)	92.90<br>(21.28)	9.43<br>(1.74)	4.96<br>(1.58)

Each group of mice was first colonized by *S. gordonii* DL1. Values are average of five pools (standard deviation). For each organic acid there are no differences between groups at 95% confidence by one-way ANOVA with Sidak's multiple comparisons test. 3HBA, 3-hydroxybutyric acid

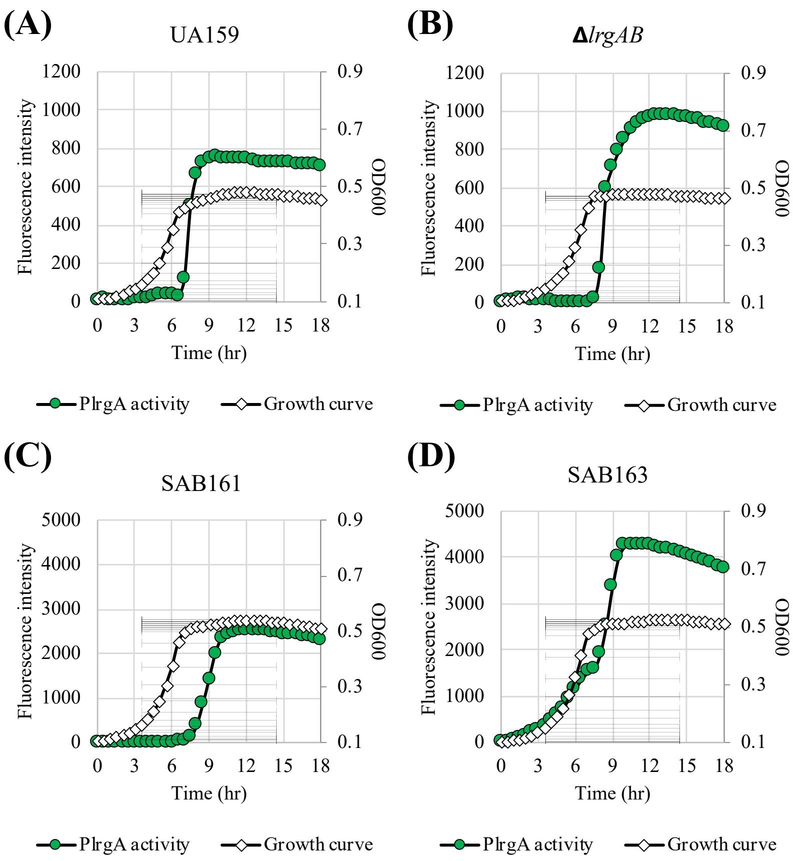

Supplementary Figure 1. Change of P*lrgA* activity over the growth in the *lrg*-derivative strains. The *S. mutans* UA159 (A), Δ*lrgAB* (B), SAB161 (C), and SAB163 (D) strains, harboring the P*lrgA*-*gfp* construct in pDL278, were grown in FMC medium supplemented by 11 mM glucose

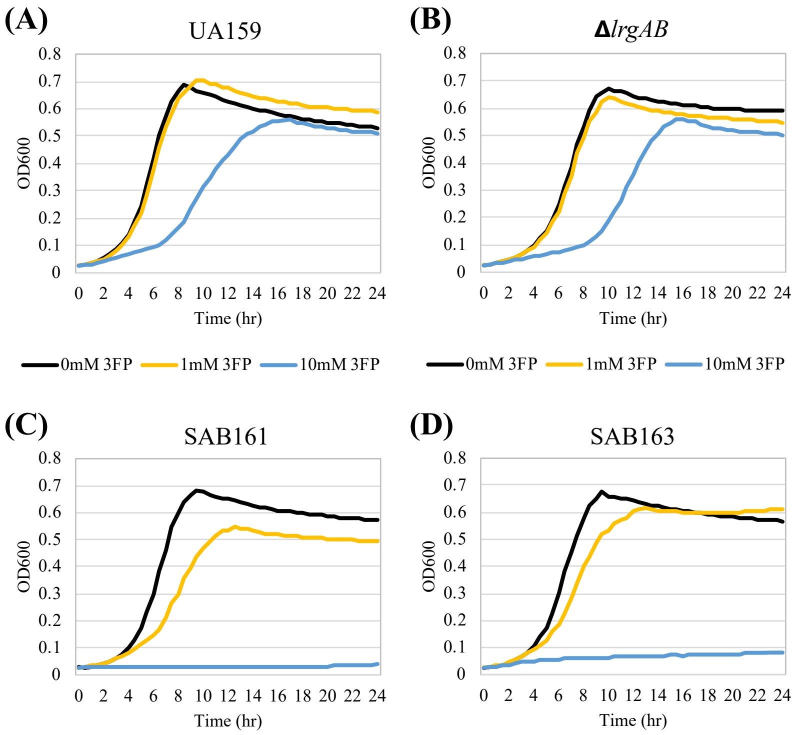

Supplementary Figure 2. The effect of 3-fluoropyruvate (3FP; pyruvate analogue) on the growth of the *lrg*-derivative strains in TV medium. The strains, including *S. mutans* UA159 (WT, A), Δ*lrgAB* (*lrgAB*-deficient, B), SAB161 (*lrgAB*-overexpressing, C), and SAB163 (*lytST*-overexpressing, D), were cultivated in TV (tryptone/vitamin) medium supplemented by different concentrations of 3FP (0, 1, and 10 mM).

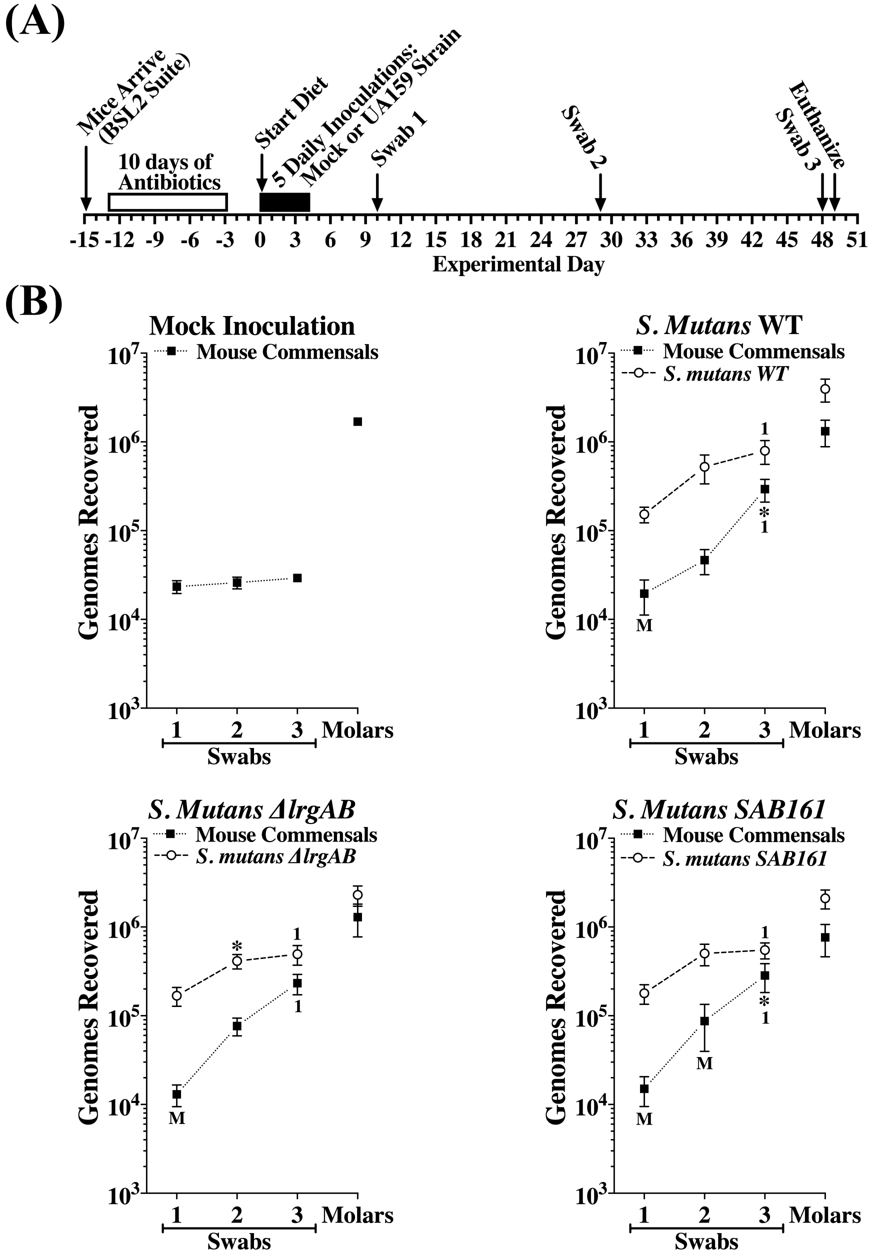

Supplementary Figure 3. Colonization of the oral cavity and mandibular molars of mice by wild type *S. mutans* UA159 (WT), Δ*lrgAB* (*lrgAB*-deficient) and SAB161 (*lrgAB*-overexpressing) strains. A, Timeline of key events in the experiment; B, Colonization results for each indicated inoculated strain and mouse oral commensals (Commensals) from oral swabs 1-3 and from sonicates of mandibular molars taken at the times indicated in (A). Statistical comparisons by one-way ANOVA with Tukey-Kramer multiple comparisons test. *, $p \leq 0.05$ versus the previous swab or an earlier swab as indicated by the swab number, or versus the same point in the mock (M) group.

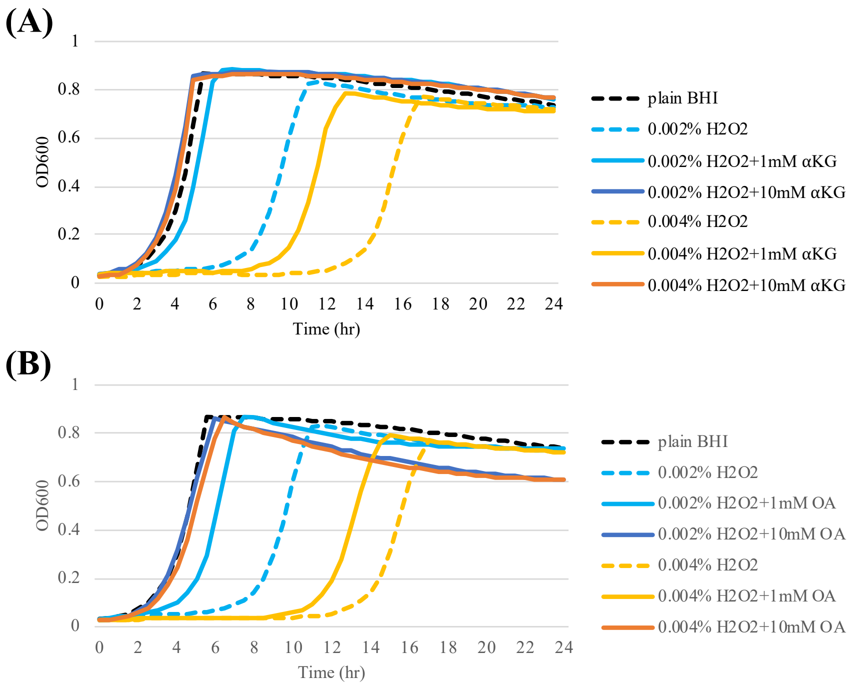

Supplementary Figure 4. H_2O_2-scavenging activity of α-ketoglutarate (αKG), and oxaloacetate (OA). *S. mutans* UA159 (WT) were cultivated in BHI medium, supplemented by H_2O_2 (0.002% and 0.004%), and αKG (0, 1 and 10 mM, A) or OA (0, 1 and 10 mM, B).
